# Stereochemically Active Lone Electron Pairs in the Chalcogen Oxide-Carbonates Te[CO_3_]O and Te_4_O_7_[CO_3_] at Elevated Pressures

**DOI:** 10.1021/acs.inorgchem.6c02651

**Published:** 2026-07-17

**Authors:** Dominik Spahr, Lkhamsuren Bayarjargal, Ninel Sharapova, Valentin Kovalev, Andrey Aslandukov, Fariia I. Akbar, Elena Bykova, Maxim Bykov, Victor Milman, Nico Giordano, Björn Winkler

**Affiliations:** † 9173 Goethe University Frankfurt, Institute of Geosciences, Altenhöferallee 1, 60438 Frankfurt, Germany; ‡ Stony Brook University, Department of Geosciences, 255 Earth & Space Science Building, Stony Brook, New York 11794-2100, United States; § Goethe University Frankfurt, Institute of Inorganic and Analytical Chemistry, Max-von-Laue-Straße 7, 60438 Frankfurt, Germany; ∥ Dassault Systèmes BIOVIA, 22 Cambridge Science Park, Cambridge CB4 0FJ, United Kingdom; ⊥ Deutsches Elektronen-Synchrotron DESY, Notkestrasse 85, 22607 Hamburg, Germany

## Abstract

Two anhydrous tellurium carbonates, Te­[CO_3_]O and Te_4_O_7_[CO_3_], have been synthesized in laser-heated diamond anvil cells by a reaction of TeO_2_ with CO_2_. Te­[CO_3_]O was obtained at higher pressures (40(2) GPa), while Te_4_O_7_[CO_3_] was synthesized at moderate pressures (20(2) GPa). The crystal structures of both compounds were determined from synchrotron single crystal X-ray diffraction data and confirmed by density functional theory-based calculations. For Te­[CO_3_]O the experimental Raman data were accurately reproduced by a Raman spectrum derived from the calculations. Both carbonates belong to the family of sp^2^-carbonates and are characterized by the presence of trigonal-planar [CO_3_]^2–^-groups. While the crystal structure of Te­[CO_3_]O is rather simple, Te_4_O_7_[CO_3_] is a layered compound with layers of [CO_3_]^2–^-groups alternating with complex Te–O-layers. Our DFT-based calculations show that stereochemically active lone electron pairs are present in both crystal structures at elevated pressures. The successful synthesis of Te­[CO_3_]O is a significant enlargement of the crystal chemistry of carbonates, as it demonstrates that anhydrous carbonates of chalcogenide group elements hosting no further cations can be synthesized. Excluding the noble gases, the chalcogenide group was the last main group of the periodic table where such carbonates were not known up to now.

## Graphical abstract

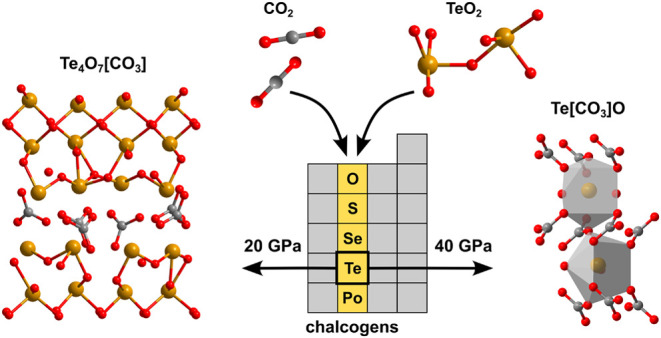

## Introduction

The crystal chemistry of chemically simple anhydrous sp^2^-carbonates attracted significant attention in the last years due to a growing number of high-pressure experiments. Such “conventional” carbonates are characterized by trigonal-planar [CO_3_]^2–^-groups as their defining structural building blocks in which sp^2^-hybrid orbitals are present between the central carbon atom and the surrounding oxygen atoms, and contain only one type of cation.^1,2^ Until recently, such carbonates were limited to those containing alkali and alkaline earth cations (e.g., Na_2_[CO_3_] or Ca­[CO_3_]) or 3*d* transition metal cations (e.g., Mn­[CO_3_] to Zn­[CO_3_] and Cd­[CO_3_]).^3–9^ A great step forward for understanding the crystal chemistry of carbonates was the synthesis of Be­[CO_3_], the last missing member of the alkaline earth metal carbonates, at moderate pressures and the discovery that its high-pressure polymorph is isostructural to Mg­[CO_3_],^10,11^ following the “pressure-homologue rule”.^12^

The next significant expansion of the crystal chemistry of sp^2^-carbonates was the discovery that chemically simple carbonates hosting M^3+^ metal cations such as Al_2_[CO_3_]_3_, Fe_2_[CO_3_]_3_ or Cr_2_[CO_3_]_3_ can be obtained at moderate pressures.^13–15^ Moreover, Al_2_[CO_3_]_3_ was the first representative of a carbonate containing an element from the boron group. The same experimental approach also allowed the synthesis of the anhydrous carbonates U­[CO_3_]_2_ and Ti_2_O_3_[CO_3_] hosting M^4+^ metal cations.^16,17^ The very recent description of the first carbonate of silicon, Si­[CO_3_]_2_, demonstrated unambiguously that not only metals but also metalloid cations can form chemically simple carbonates.^18^ The latest enlargement of the carbonate chemistry was the synthesis of (IO_2_)_2_[CO_3_].^19^ This di-iodyl carbonate hosts nonmetal I^5+^ cations and it was the first compound containing only a halogen group element as a cation in a carbonate. This discovery was specifically of interest after the synthesis of the mixed-anion iodide carbonate Na_5_(CO_3_)_2_I, where iodine is not present as a cation but I^–^ anions are present next to [CO_3_]^2–^-groups.^20^

In contrast to carbonates with cations from groups 1–15 and 17, no chemically simple carbonate or oxide-carbonates of a chalcogenide has been characterized up to now. Therefore, a timely and relevant question is whether a chalcogen can be incorporated into carbonates as the only cation species. Here, we address this question by investigating tellurium as a potential cation. Tellurium is a metalloid and known to occur as a cation (e.g., in Te^IV^O_2_ or Te^VI^O_3_), as well as an anion (e.g., in Sb_2_Te_3_, PbTe or AuTe_2_) with different oxidation states.^21–25^ This flexibility makes it a promising candidate to form a compound hosting [CO_3_]^2–^-groups. Moreover, in the naturally occurring mineral mroseite (CaTeO_2_[CO_3_]) the tellurium atoms are present as a cation, but next to calcium atoms.^26^ In the complex dialkyltelluroxane carbonates (Me_2_TeOTeMe_2_[CO_3_])_*n*_ or HO­(CH_2_)_4_TeOTe­(CH_2_)_4_[CO_3_]­Te­(CH_2_)_4_OH·H_2_O further H_2_O, CH_3_ or (CH_2_)_4_ groups are stabilizing the crystal structure.^27^ Hence it is unclear, if a chemically simple carbonate of tellurium i. e. of a chalcogenide can be synthesized.

Tellurium compounds are of general interest due to the presence of stereochemically active lone electron pairs. The presence of such lone electron pairs significantly influences the crystal structure and determines their chemical and physical properties to a significant extent. Hence, characterizing such compounds is of great interest for inorganic chemistry.^28–31^ For example, it is well established that tellurites hosting Te^4+^ cations have in most cases stereoactive 5s^2^ lone electron pairs and that the nonlinear optical properties of compounds such as TeO_2_ are affected by these lone electron pairs.^32–34^ We have demonstrated in the past that density functional theory (DFT) based calculations can be employed to visualize the stereochemically active lone electron pairs (e.g., in Bi_2_Ga_4_O_9_, in Pb_2_SnO_4_ and in Sb_2_O_2_[CO_3_]) at elevated pressures.^35–37^

In order to understand if an anhydrous carbonate of tellurium can be synthesized in analogy to the halogen carbonate (IO_2_)_2_[CO_3_] or the chemically simple carbonates such as Be­[CO_3_], Al_2_[CO_3_]_3_ or U­[CO_3_]_2_ we investigated the reaction between TeO_2_ and CO_2_ at pressures of 20 GPa and of 40 GPa.^10,11,13,19^ In TeO_2_ four-valent Te^4+^ cations are present with an ionic radius (*r*(Te^4+^) = 0.97 Å) similar to those often observed for the cations in “conventional” calcite-type carbonates such as Ca­[CO_3_] (*r*(Ca^2+^) = 1.00 Å). The ionic radius of Te^4+^ is nearly identical to those of I^5+^ (*r*(I^5+^) = 0.95 Å) in (IO_2_)_2_[CO_3_] and slightly larger than of U^4+^ (*r*(U^4+^) = 0.89 Å) in U­[CO_3_]_2_.^6,9,16,19,38^

## Results and Discussion

The high-pressure experiments were carried out in laser-heated diamond anvil cells (LH-DACs). In a first step, a commercially available TeO_2_ powder was compacted between a glass plate and a diamond and then placed on the culet of the bottom diamond of the DAC. In a second step, cryogenic loading was employed to condense CO_2_–I (dry ice) directly into the sample chamber of the DAC (see SI). The DAC was cooled down to ≈100 K and CO_2_ was condensed into the sample chamber of the DAC until the chamber was filled and the TeO_2_ power compact was completely covered. In the last step, the DAC was closed tightly and compressed to the target pressure of the experiment without intermediate heating. After the cryogenic loading CO_2_–I is present in the sample chamber. During cold compression of the DAC a single phase transition from CO_2_–I (*Pa*3̅) to CO_2_–III (*Cmca*) occurs in a broad (≈5 GPa) pressure range around ≈12 GPa.^39–41^ At 20 GPa as well as at 40 GPa CO_2_–III is present before the laser heating. At pressures above ≈12 GPa and ≤40 GPa heating CO_2_–III will result in the formation of the high-temperature polymorphs CO_2_–II or CO_2_–IV, depending on the temperature.^41–43^ Above ≈25 GPa also CO_2_–V (*I*4̅2*d*) is present, which is the stable CO_2_-polymorph up to very high pressures (>100 GPa).^41,44^

In the first experiment the TeO_2_ + CO_2_ mixture was compressed to 40(2) GPa without intermediate heating (Figure 1 a) and laser-heated from both sides up to a maximum temperature of ≈1600(200) K (Figure 1b). The heating time was 30 min. After the heating, metastable CO_2_–III is still present around the heated area in the sample chamber (Figure 1b). In addition, we could observe the characteristic Raman modes of CO_2_–V in the laser heated area (Figure 1d). The experimental Raman spectrum of CO_2_–V is in very good agreement with the one derived from our DFT-based calculations (Figure 2a). In addition, we observed some minor Raman peaks of the low-pressure high-temperature CO_2_ polymorph II. (Figure 2a). In order to determine if a reaction between TeO_2_ and CO_2_ had occurred we employed spatially resolved Raman spectroscopy on a grid across the sample chamber. We found that an unknown phase is present in the sample chamber after heating (Figure 1c,e,f), which shows a strong Raman mode at ≈1140 cm^–1^ (Figure 2b). Raman modes occurring at this wavenumber are characteristic for the C–O stretching mode in a [CO_3_]^2–^-group of “conventional” sp^2^-carbonates hosting cations with similar ionic radius such as Mg­[CO_3_] or Ca­[CO_3_].^45,46^ In addition, our Raman maps (Figure 1e,f) reveal that a noticeable Raman mode at ≈1600 cm^–1^ (Figure 2b) belongs to the same phase. A significant Raman signal at such wavenumbers, indicating the presence of a [CO_3_]^2–^-group, was also observed in (IO_2_)­[CO_3_].^19^ From the distribution of the of the new phase in the Raman map across the heated area we infer that most of the TeO_2_ powder reacted with the CO_2_ during the formation of the unknown carbonate phase.

**Figure 1:**
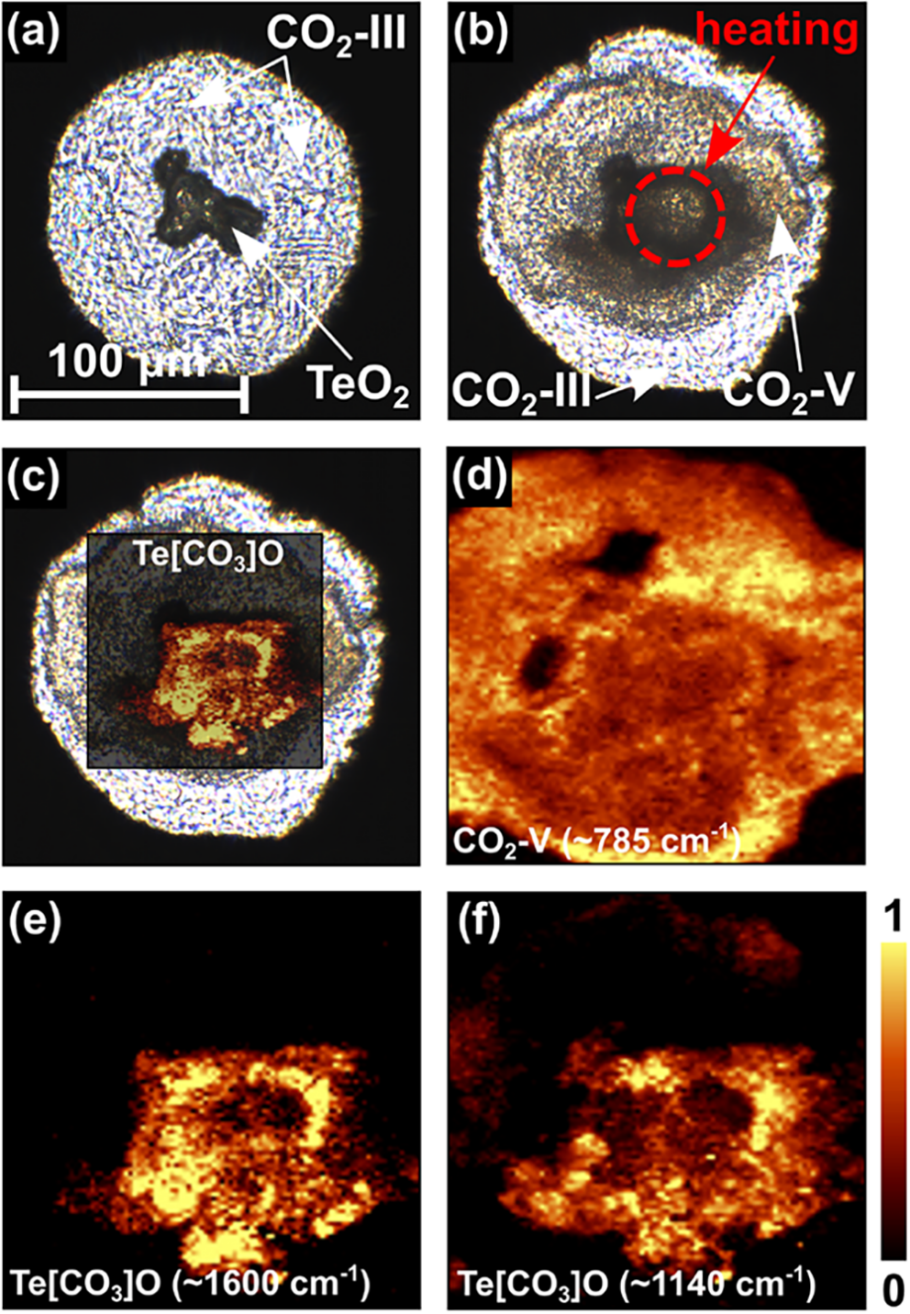
(a) Unheated TeO_2_ powder in the sample chamber of the DAC together with CO_2_–III at 40(2) GPa. (b) TeO_2_ + CO_2_ mixture after laser heating to ≈1600(200) K at 40(2) GPa. (c) Raman map of Te­[CO_3_]O after laser heating overlaid on a picture of the sample chamber. (d) Raman map of CO_2_–V (≈785 cm^–1^). Raman maps of different Te­[CO_3_]O Raman modes: (e) ≈1600 cm^–1^ and (f) ≈1140 cm^–1^.

**Figure 2:**
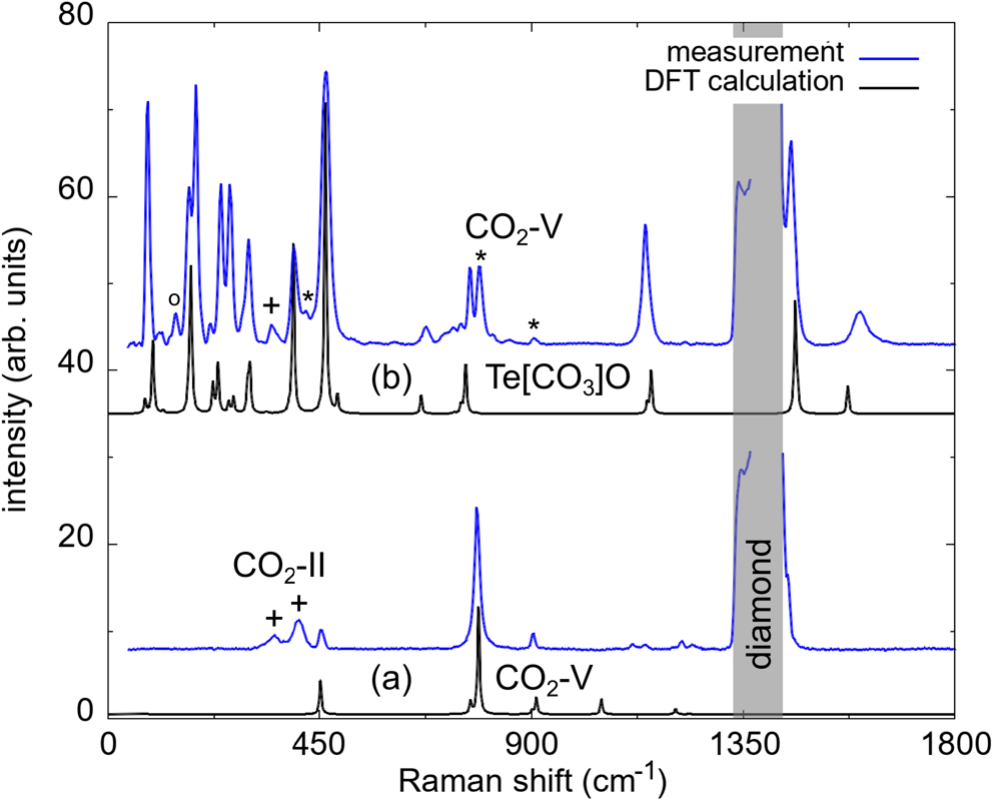
Raman spectroscopy at 40(2) GPa after laser heating the TeO_2_ + CO_2_ mixture: (a) Raman spectra for CO_2_–V. (b) Raman spectra for Te­[CO_3_]­O. Experimental Raman spectra are shown in blue, DFT-based calculations are shown in black. The Raman shifts of the theoretical spectra were scaled by 1–3%. Peaks of CO_2_–II in the Raman spectrum of CO_2_–V are marked by a cross (+). Peaks of CO_2_–V in the Raman spectrum of Te­[CO_3_]O are marked by an asterisk (*) and of CO_2_–II by a cross (+). A small Raman peak at ≈200 cm^–1^ could not be assigned to Te­[CO_3_]O or any CO_2_ polymorph. The Raman mode is marked by a circle (°).

In order to determine the crystal structure of the unknown phase we used synchrotron-based X-ray diffraction. First, X-ray powder diffraction data were collected on a 2D grid across the sample chamber. Afterwards we collected diffraction data suitable for single crystal X-ray diffraction on selected grid positions with unidentified reflections. The results from the single crystal structure solution and refinement show that the unknown phase is an anhydrous and chemically simple tellurium oxide-carbonates with Te­[CO_3_]O composition. Te­[CO_3_]O crystallizes in the monoclinic space group *P*2_1_/*c* with *Z* = 4 (Figure 3a). The lattice parameters at 40(2) GPa are *a* = 4.3690(3) Å, *b* = 5.606(2) Å, *c* = 8.4270(8) Å and β = 90.32(1)° (*V* = 206.41(7) Å^3^). The very low *R*_1_-value of 3.0% and the high reflection-to-parameter-ratio (10.5:1) are indicative of a very good structure refinement. In addition, the displacement parameters of the light carbon and oxygen atoms could be refined anisotropically next to the heavy tellurium atoms, confirming the very high quality of the collected diffraction data. No constraints or restraints had to be introduced for the structure refinement. The experimental structural model is in very good agreement with the one derived from our DFT-based full geometry optimization (Table S1). Moreover, the experimental Raman spectrum is accurately reproduced by a theoretical Raman spectrum obtained from our DFPT-calculations (Figure 2b), confirming that the structural model is appropriate (*P*2_1_/*c*). There are 36 Raman active modes. However, we noticed, that the β-angle is very close to 90° (SC-XRD: 90.32(1)°), hence we tested the experimental structural model using the PLATON/checkCIF program in order to check for a higher space group symmetry.^47^ PLATON/checkCIF suggests the orthorhombic space group *Pbcm* as a possible higher symmetry. Performing a reasonably successful structure solution and refinement in space group *Pbcm* was not possible. Due to symmetry constraints on the tellurium atom position the *R*_1_-value is very high for a refinement in the orthorhombic space group (≥20%). Hence, we think that the monoclinic (*P*2_1_/*c*) structural model for Te­[CO_3_]O is appropriate for our experimental data.

**Figure 3:**
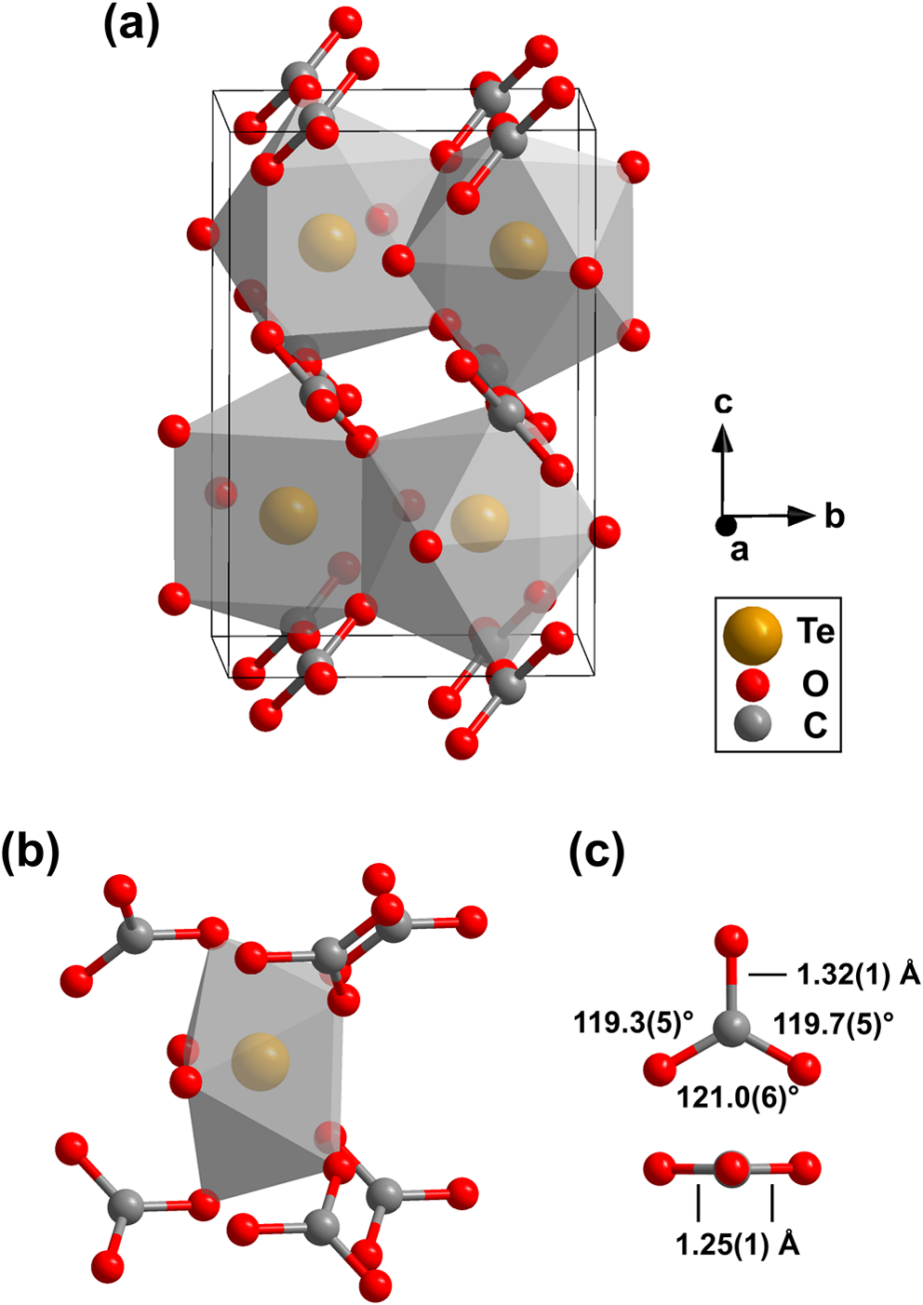
(a) Crystal structure of Te­[CO_3_]O obtained from synchrotron single-crystal X-ray diffraction at 40(2) GPa (*P*2_1_/*c*, *Z* = 4). (b) Coordination polyhedron of the tellurium atom by oxygen. (c) Geometry of the [CO_3_]^2–^-group from single-crystal X-ray diffraction.

Te­[CO_3_]O is a chemically simple oxide-carbonate. It belongs to the family of anhydrous *sp* ^2^-carbonates hosting [CO_3_]^2–^-groups as central building blocks (Figure 3). The [CO_3_]^2–^-groups are nearly ideally trigonal-planar with C–O bond distances of 1.25(1) Å and 1.32(1) Å (Figure 3c). The experimental O–C–O angles are all very similar 119.3(5)°, 119.7(5)° and 121.0(6)°. The C–O bond distances (1.25 Å and 1.30 Å) and O–C–O angles (119.4° and 120.3°) derived from our DFT-based calculations reproduce the experimental values with the expected uncertainties. The Mulliken population analysis yielded nearly identical values for the C–O bonds (0.94 e^–^/Å^3^ and 0.79 e^–^/Å^3^) and suggests a strong covalent bonding within the [CO_3_]^2–^-group. The remaining oxygen atom, which is not part of the [CO_3_]^2–^-group, is only connected to two tellurium atoms. The experimental (1.93(1) Å) and calculated (2.00 Å) Te–O bond distances match well. Each tellurium atom is coordinated by 8 oxygen atoms (Figure 3b). Two of these oxygens are not part of a [CO_3_]^2–^-group. In contrast to the complex dialkyltelluroxane carbonates (Me_2_TeOTeMe_2_[CO_3_])_*n*_ or HO­(CH_2_)_4_TeOTe­(CH_2_)_4_[CO_3_]­Te­(CH_2_)_4_OH·H_2_O no further H_2_O, CH_3_ or (CH_2_)_4_ groups are required to stabilize the crystal structure of Te­[CO_3_]­O.^27^ The geometry of the [CO_3_]^2–^-groups in the complex dialkyltelluroxane carbonates or in mroseite are in agreement with the geometry derived for the [CO_3_]^2–^-group in Te­[CO_3_]O at elevated pressures hosting no further cations or building blocks.^26,27^

In a second experiment the TeO_2_–CO_2_ system was investigated at lower pressures (20(2) GPa), in order to understand if Te­[CO_3_]O can also be obtained at significantly lower pressures. From our single crystal X-ray diffraction data collected at 20(2) GPa we found that Te­[CO_3_]O could not be synthesized at this pressure. However, our data clearly show that a reaction between TeO_2_ and CO_2_ had occurred. We solved the crystal structure of the second tellurium carbonate, which was present across the heated area in the sample chamber of the DAC. The second anhydrous carbonate phase at 20(2) GPa has Te_4_O_7_[CO_3_] composition. The oxide-carbonate Te_4_O_7_[CO_3_] crystallizes in the acentric orthorhombic space group *Iba*2 with *Z* = 8 (Figure 4a). The lattice parameters are *a* = 21.576(1) Å, *b* = 10.671(2) Å and *c* = 5.5405(2) Å (*V* = 1275.6(2) Å^3^). The crystal structure refinement is robust (*R*_1_-value = 3.3% and reflection-to-parameter-ratio = 15.8:1). The displacement parameters of all 16 symmetrically independent atoms could be refined anisotropically without introducing any constraints or restraints, confirming the very high quality of the collected diffraction data. In addition, our DFT-based full geometry optimization confirmed the experimental structural model (Table S2). Our XRD data show the presence of remaining acentric TeO_2_-*P*2_1_2_1_2_1_, preventing an unambiguous detection of the second harmonic generation (SHG) signal of Te_4_O_7_[CO_3_]-*Iba*2.

**Figure 4:**
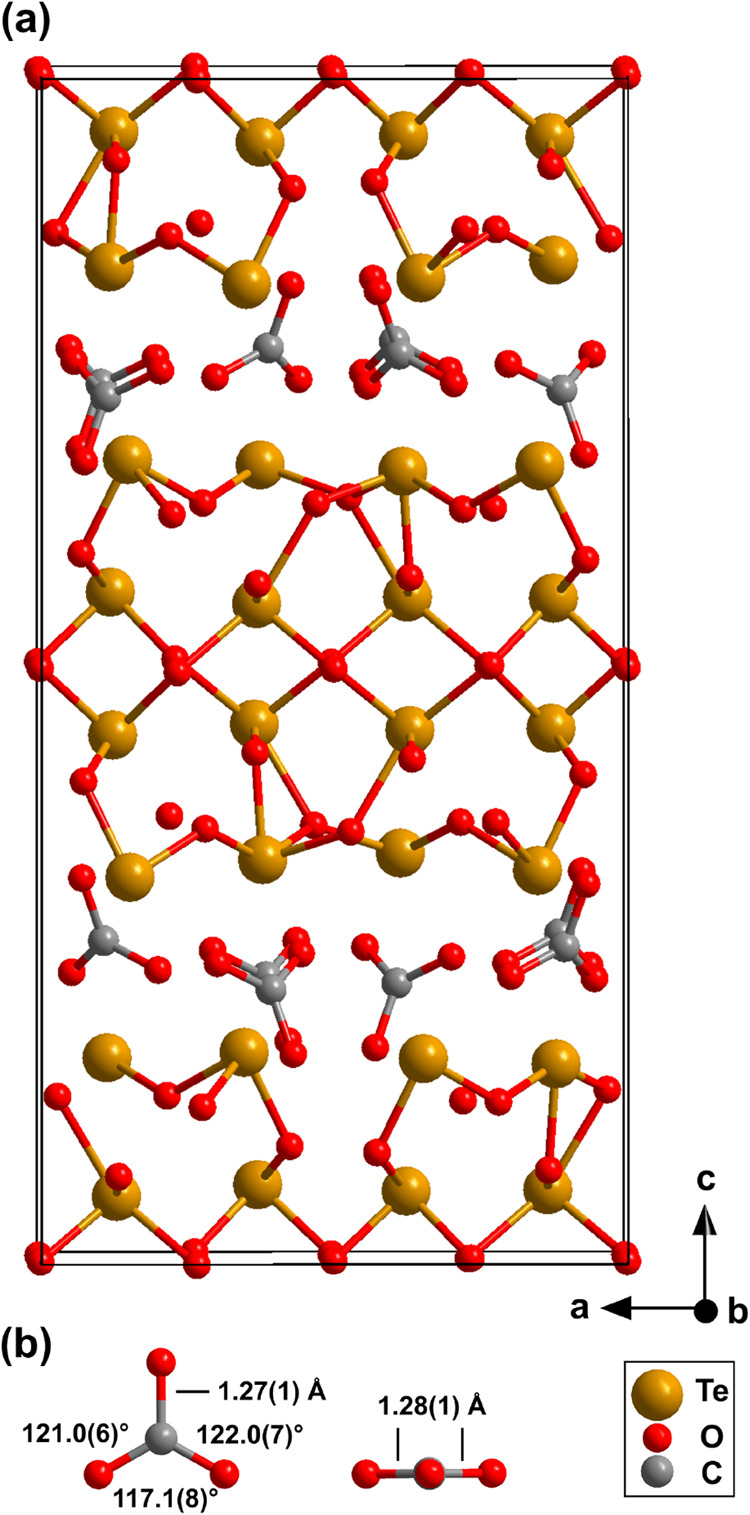
(a) Crystal structure of Te_4_O_7_[CO_3_] obtained from synchrotron single-crystal X-ray diffraction at 20(2) GPa (*Iba*2, *Z* = 8). (b) Geometry of the [CO_3_]^2–^-group from single-crystal X-ray diffraction.

Te_4_O_7_[CO_3_] belongs also to the family of anhydrous sp^2^-carbonates, in particular to the subgroup of oxide-carbonates. It has a complex layered crystal structure (Figure 4a). One layer consists of trigonal-planar [CO_3_]^2–^-groups (Figure 4b). Similar to Te­[CO_3_]O the [CO_3_]^2–^-groups in Te_4_O_7_[CO_3_] are nearly ideal trigonal-planar with C–O bond distances between 1.27(1) Å and 1.28(1) Å and O–C–O angles between 117.1(8)° and 122.0(7)°. The C–O bond distances (1.25 Å to 1.23 Å) and O–C–O angles are in good agreement with our DFT-calculations. Our Mulliken population analysis reveals strong covalent C–O bonds (0.95 e^–^/Å^3^ to 0.79 e^–^/Å^3^) within the [CO_3_]^2–^-groups. The Mulliken populations are in agreement with the populations derived for Te­[CO_3_]O at 40 GPa. The second layer of the Te_4_O_7_[CO_3_] crystal structure consists of a complex network of tellurium and oxygen atoms with “Te_4_O_7_”-composition. All remaining oxygen atoms, which are not part of the [CO_3_]^2–^-groups, are involved. The Mulliken population analysis of the Te–O bonds within the layer yields significantly lower values (between 0.16 e^–^/Å^3^ and 0.30 e^–^/Å^3^) than for the C–O bonds. The interactions between the tellurium atoms within the layer and the oxygen atoms of the [CO_3_]^2–^-groups are even weaker (≤0.10 e^–^/Å^3^). It is worthwhile to note that in the TeO_2_–SO_3_ system compounds such as TeO­[SO_4_], Te_2_O_3_[SO_4_] and Te_4_O_3_[SO_4_]_5_ with a similar tellurium–oxygen sublattice have been found.^22,48^ Hence, it is likely that also in the TeO_2_–CO_2_ system further novel carbonates with different tellurium–oxygen sublattices can be synthesized.

We employed our DFT-based calculations to investigate the electron density distribution in the crystal structure of Te­[CO_3_]­O. The calculations clearly show lone electron pairs as umbrella shaped maxima in the electron difference density map, ≈0.7 Å away from the tellurium atom (Figure 5a). The lone electron pairs are stereochemically active. Such stereochemically active lone electron pairs have earlier been observed e. g. in Bi_2_Ga_4_O_9_, in Pb_2_SnO_4_ and in Sb_2_O_2_[CO_3_] at elevated pressures.^35–37^ The existence of the lone electron pairs of the tellurium atom results in an asymmetric shape of the Te–O coordination polyhedron in Te­[CO_3_]O (Figure 3b). We performed the same analysis for the crystal structure of Te_4_O_7_[CO_3_]. Similar to Te­[CO_3_]O our DFT calculations clearly show lone electron pairs as umbrella shaped maxima in the electron difference density map (Figure 5b). Both compounds are insulators. The DFT-GGA-PBE calculations yield weakly pressure-dependent band gaps of ≈2.3 eV for Te_4_O_7_[CO_3_] and ≈2.7 eV for Te­[CO_3_]­O, but it is well established that these values will be significantly underestimated.

**Figure 5:**
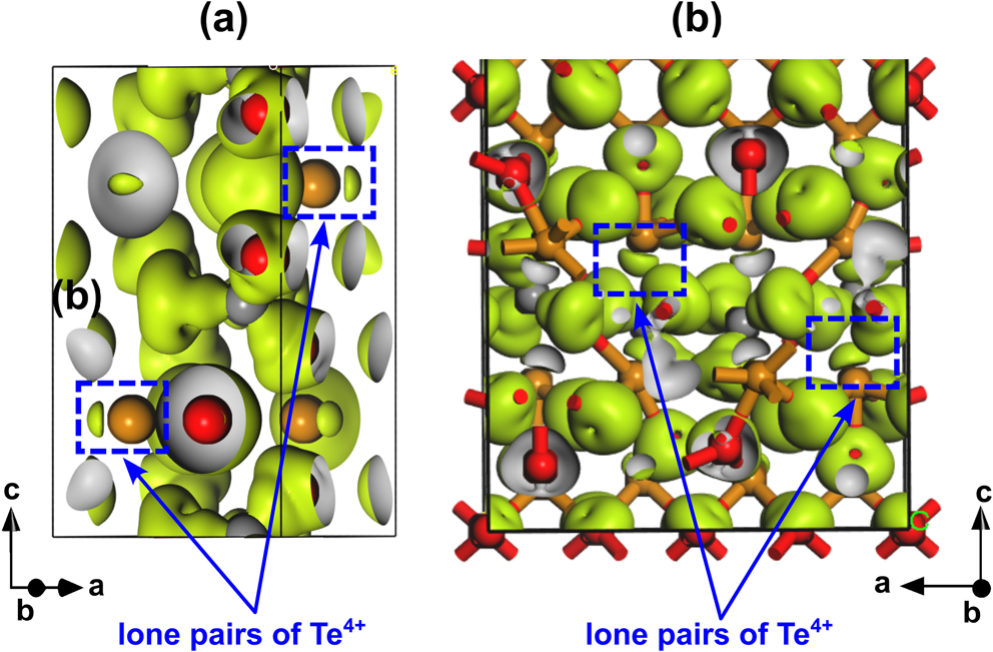
Electron difference density map (a) of Te­[CO_3_]O and (b) of Te_4_O_7_[CO_3_] from DFT-based calculations. The stereochemically active lone electron pairs in both crystal structures manifest themselves as umbrella shaped maxima in the map, about 0.7 Å away from the Te^4+^ cation. The isosurface shown represents electron difference densities of 0.04 e^–^/Å^3^.

After we established that our DFT-calculations accurately reproduce the experimentally determined structures of Te­[CO_3_]O as well as of Te_4_O_7_[CO_3_] at their synthesis pressures, we employed further calculations to derive the *p*,*V*-relation for both phases. From the calculations we predict that Te­[CO_3_]O is undergoing an isostructural phase transition during decompression. The calculated *p*,*V*-data of the different Te­[CO_3_]O phases were fitted separately with an equation of state (EoS) in order to determine the bulk modulus *K*_0_ and its pressure derivative *K*_p_ (Figure S3). We obtained a bulk modulus of *K*_0_ = 80.0(6) GPa with *K*_p_ = 5.76(4) for the high-pressure phase of Te­[CO_3_]O and a significantly lower bulk modulus of *K*_0_ = 32.4(6) GPa with *K*_p_ = 6.2(1) for its low-pressure phase. The ambient pressure bulk modulus of the high-pressure phase is in the same range than reported for other “conventional” carbonates hosting medium sized cations, such as Ca­[CO_3_] (67(2) GPa) or Mg­[CO_3_] (107(1) GPa).^49^ For Te_4_O_7_[CO_3_] we derived a bulk modulus of *K*_0_ = 51(4) GPa with *K*_p_ = 6.7(4) (Figure S4). This value is in between the bulk modulus derived for the low- and high-pressure phase of Te­[CO_3_]­O. The pressure-dependent compressibility *K*_p_ is relatively large in comparison with other “conventional” carbonates. However, the values are similar to the ones derived for (IO_2_)_2_[CO_3_] at similar pressures.^19^

## Conclusion

In summary we have synthesized two anhydrous carbonates of tellurium at elevated pressures, Te­[CO_3_]O (40(2) GPa), and Te_4_O_7_[CO_3_] (20(2) GPa), by a reaction of TeO_2_ with CO_2_. Both belong to the subgroup of oxide-carbonates. Single crystal X-ray diffraction was employed to determine the crystal structure of both phases after their synthesis inside the LH-DAC. DFT-based calculations were used to confirm the experimental structural models of both compounds. The crystal structures of both phases are characterized by trigonal-planar [CO_3_]^2–^-groups and hence Te­[CO_3_]O and Te_4_O_7_[CO_3_] belong to the family of anhydrous sp^2^-carbonates. Moreover, in both phases four-valent Te^4+^-cations are present. The stereochemically active lone electron pairs manifest themselves as umbrella shaped maxima in the electron difference density map, about 0.7 Å away from the tellurium atoms in both phases in our DFT-calculations. The successful synthesis of Te­[CO_3_]O answers the question if an anhydrous carbonate of a chalcogenide group element, hosting no further cations, can be synthesized. This is a significant extension of the crystal chemistry of carbonates as, neglecting the noble gases, the chalcogenide group was the last main group of the periodic table where such an anhydrous carbonate was not known up to now. In addition, after the recent synthesis of Si­[CO_3_]_2_,^18^ the synthesis of Te­[CO_3_]O confirms that metalloid cations can form chemically simple carbonates or oxide-carbonates. Moreover, we are confident that the synthesis of Te­[CO_3_]O as well as of Te_4_O_7_[CO_3_] provides a roadmap for the synthesis of further novel carbonates hosting metalloid or nonmetal cations e. g. of chalcogen group elements such as selenium or sulfur. This would provide a broader basis for a systematic crystal chemistry of anhydrous metalloid or nonmetal carbonates. In addition, it would facilitate the investigation of compounds characterized by the presence of lone electron pairs at elevated pressures, which in turn may have interesting chemical and physical properties.^28–34,50^

## Methods

Commercial tellurium dioxide (TeO_2_) powder was used as starting material for the high-pressure experiments (99.995% purity, Merck KGaA, Darmstadt, Germany). The high-pressure experiments were carried out in Boehler-Almax type diamond anvil cells (DACs).^51^ CO_2_ (Nippon gases, purity ≥99.996%) was loaded cryogenically into into the DAC as dry ice by a custom-built cryogenic loading system.^19^ The TeO_2_ powder was laser-heating from both sides using a custom-built setup equipped with a Coherent Diamond K-250 pulsed CO_2_ laser (λ = 10600 nm) at the target pressures of the different experiments.^46^ For high-pressure Raman spectroscopy, we employed an Oxford Instruments WITec α 300R Raman imaging microscope equipped with an Olympus SLMPan N 50× objective. The measurements were performed using the 532 nm laser and the 1200 grooves mm^–1^ grating of the WITec UHTS 300S (VIS-NIR) spectrograph. High-pressure single-crystal synchrotron X-ray diffraction experiments were carried out at PETRA III (DESY) in Hamburg, Germany, at the extreme conditions beamline P02.2 and at the ESRF in Grenoble, France, at the Materials Science Beamline line ID11.^52,53^ First-principles calculations were carried out within the framework of density functional theory (DFT), employing the Perdew–Burke–Ernzerhof (PBE) exchange-correlation functional and the plane wave/pseudopotential approach implemented in the CASTEP simulation package.^54–56^ In addition, we employed a correction scheme for van der Waals (v.d.W.) interactions.^57^ A detailed description of the experimental and computational methods is available in the Supporting Information.

## Supplementary Material



## Notes

Experimental and computational details of the experiments and the results of the single-crystal structure solution and DFT-based calculations (PDF)
