# Supplementary material for: Stereochemically Active Lone Electron Pairs in the Chalcogen Oxide-Carbonates Te[CO3]O and Te4O7[CO3] at Elevated Pressures
Source: Inorg Chem. 2026 Jul 17;65(30):17749–55. doi: 10.1021/acs.inorgchem.6c02651 (PMC13439661; doi:10.1021/acs.inorgchem.6c02651)
Supplement: Supplementary file 1 [file ic6c02651_si_001.pdf]

Supporting Information: Stereochemically active lone electron pairs in the chalcogen oxide-carbonates $Te[CO_3]O$ and $Te_4O_7[CO_3]$ at elevated pressures

Dominik Spahr[*,a,b], Lkhamsuren Bayarjargal[a], Ninel Sharapova[a], Valentin Kovalev[a], Andrey Aslandukov[c], Fariia I. Akbar[c], Elena Bykova[a], Maxim Bykov[c], Victor Milman[d], Nico Giordano[e], Björn Winkler[a]

[a]*Goethe University Frankfurt, Institute of Geosciences, Altenhöferallee 1, 60438 Frankfurt, Germany*
[b]*Stony Brook University, Department of Geosciences, 255 Earth & Space Science Building, Stony Brook, NY 11794-2100, USA*
[c]*Goethe University Frankfurt, Institute of Inorganic and Analytical Chemistry, Max-von-Laue-Straße 7, 60438 Frankfurt, Germany*
[d]*Dassault Systèmes BIOVIA, 334 Cambridge Science Park, Cambridge CB4 0WN, United Kingdom*
[e]*Deutsches Elektronen-Synchrotron DESY, Notkestrasse 85, 22607 Hamburg, Germany*

1. Methods

1.1. *Sample material*

We used commercial tellurium dioxide (TeO_2) powder as starting material for the high-pressure experiments (99.995% purity, Merck KGaA, Darmstadt, Germany). The TeO_2 powder was used as received without further purification. Before the loading of the diamond anvil cells (DACs) the TeO_2 powder was compacted between a diamond and a glass plate. The CO_2 gas for the gas-jet was used as purchased (Nippon gases, purity $\geq$ 99.996%).

1.2. *High-pressure experiments*

The high-pressure experiments, with different target pressures, were carried out using Boehler-Almax type DACs.[1] For the experiment at 40 GPa as well as for the one at 20 GPa the DACs were equipped with diamonds having an opening angle of 70° and 300 µm sized culets on both sides. We used a Re-gasket, which were pre-indented to thicknesses between $\approx$ 40–45 µm in our experiments. We employed a custom-built laser set-up to drill sample chambers with $\approx$ 120 µm diameter into the pre-indented Re-gaskets. The pressure during compression of the DACs was derived from the position of the high frequency edge of the diamond Raman band and we assume an error of at least 5% due to non-hydrostatic conditions in the DAC.[2] In addition, we expect that the pressure conditions in the DAC before laser heating are very likely non-hydrostatic as CO_2-III may sustain pressure gradients up to 0.2 GPa µm^{-1} at high pressures without heating.[3]

In the first step a TeO_2 powder compact, with dimensions of $\approx 50 \times 30 \times 10$ µm^3, was placed on the culet of the bottom diamond of each DAC. Afterwards, the DACs were placed on a liquid nitrogen cooled Cu-holder for the cryogenic loading and cooled down using a custom-built cryogenic loading system (see Spahr *et al.*[4]). The DACs were cooled down to $\approx$ 120 K and CO_2-I (dry ice) was directly condensed into the sample chamber from the CO_2 gas jet. We used a small nozzle to align the CO_2 gas jet with 5 l min^{-1} directly on the gap between upper diamond and the gasket. We used argon (5 l min^{-1}) as a purge gas in order to avoid the co-precipitation of H_2O in the sample chamber of the DAC. During the loading process the precipitation of the CO_2 in the sample chamber was monitored using an optical microscope and equipped with a camera. After the sample chamber was completely covered with dry-ice, the enclosure was opened and each DAC was tightly closed. In the last step, the DACs were compressed to the target pressures of the different experiments (40 GPa and 20 GPa).

1.3. *Laser heating*

In the first experiment the TeO_2 powder compact in the CO_2 environment was laser-heated at 40(2) GPa. In the second experiment the laser-heating was performed at 20(2) GPa. In both experiments the sample was laser-heated from both sides using a custom-built set-up equipped with a Coherent Diamond K-250 pulsed CO_2 laser (λ = 10600 nm).[5] The laser power was adjusted to achieve a coupling of the laser to the sample, using a laser power between 1–6 W. The maximum temperature achieved during the laser-heating was $T_{\max} \leq 1600(200)$ K. The temperatures were determined by the two-color pyrometer method, employing Planck and Wien fits.[6] The heating time at both pressures was $\approx$ 30 minutes. It is well established that laser-heating in DACs always suffers from large temperature gradients and the actual temperature is strongly dependent on the coupling of the laser with the sample, especially at lower temperatures. We estimate an uncertainty of at least $\pm$15% of the nominal temperature in the laser-heated region depending on the focus of the laser beam, based on typical 2D temperature-gradient determination experiments performed in DACs.[7]

1.4. *Raman spectroscopy*

Raman spectroscopy at high-pressures in DACs was performed using an Oxford Instruments WITec alpha 300R Raman imaging microscope. The Raman microscope was equipped with an Olympus SLMPan N 50× objective. The measurements were performed using the 532 nm laser. We employed the 1200 grooves mm^{-1} grating of the WITec UHTS 300S (VIS-NIR) spectrograph in combination with an Andor DR316B-LDC-DD CCD detector for the measurements. The laser power was 100 mW on the sample and the spot size of the Raman laser was $\approx$ 0.8 µm. We assume a depth resolution of $\approx$ 6 µm in the direction of the laser beam. Raman maps were measured on a 2D-grid with a step-size of 1 µm. The background of the

*d.spahr@kristall.uni-frankfurt.de

Raman spectra was corrected using the software package Fityk.[8]

1.5. Single-crystal synchrotron X-ray diffraction

Single-crystal synchrotron X-ray diffraction after the synthesis of $Te[CO_3]O$ at 40(2) GPa was carried out at the synchrotron PETRA III (DESY) in Hamburg, Germany, at the Extreme Conditions Beamline P02.2.[9] The beam size on the sample was $\approx 2 \times 2$ μm^2 (FWHM), focused by Kirkpatrick Baez mirrors. The diffraction data were collected using a Perkin Elmer XRD1621 detector, a wavelength of 0.2904 Å (42.7 keV) and a sample to detector distance of 400.4 mm. In contrast, the crystal structure of $Te_4O_7[CO_3]$ at 20(2) GPa was obtained from single-crystal diffraction collected at the ESRF in Grenoble, France, at the Materials Science Beamline line ID11.[10] The beam size on the sample was 0.6×0.6 μm^2 and the diffraction data were collected using an Eiger2 X 4M CdTe detector. We used a wavelength of 0.2844 Å (43.6 keV) and a sample to detector distance of 148.2 mm. In both experiments, the DACs were rotated by $\pm 32°$ around the vertical axis perpendicular to the beam while collecting frames in 0.5° steps with 0.5 s acquisition time per frame.

On both beam lines the sample to detector distance was calibrated using the powder diffraction pattern of a CeO_2 standard in conjunction with the software DIOPTAS.[11] The diffractometer/detector geometry for the analysis of the single crystal diffraction data was calibrated using diffraction data collected from a single crystal of enstatite ($MgSiO_3$) in a DAC at ambient pressure at P02.2 and using a silicon single-crystal at ambient pressure at ID11. After the data collection, the reflections were indexed and integrated employing CrysAlis^{PRO} (version 43.67a).[12] We used the Domain Auto Finder program (DAFi) to find possible single-crystal domains for the subsequent data reduction.[13] The structure solution and refinement were performed using the software package OLEX2 employing SHELXT for the crystal structure determination and SHELXL for the refinement.[14,15,16]

1.6. Density functional theory-based calculations

First-principles calculations were carried out within the framework of density functional theory (DFT), employing the Perdew-Burke-Ernzerhof (PBE) exchange-correlation functional and the plane wave/pseudopotential approach implemented in the CASTEP simulation package.[17,18,19] CASTEP and auxiliary programs, including those for a symmetry analysis, an analysis of the electron density distribution, and for visualizations, were run within the BIOVIA Materials Studio suite of programs.[20]

"On the fly" norm-conserving or ultrasoft pseudopotentials generated using the descriptors in the CASTEP data base were employed in conjunction with plane waves up to a kinetic energy cutoff of 1020 eV or 630 eV, for norm-conserving and ultrasoft pseudopotentials, respectively. The accuracy of the pseudopotentials is well established.[21] A correction scheme for van der Waals (v.d.W.) interactions was applied in the DFT-calculations. We employed the correction scheme developed by Tkatchenko and Scheffler.[22] A Monkhorst-Pack grid was used for Brillouin zone integrations.[23] We used a distance between grid points of <0.023 Å^{-1}. Convergence criteria for geometry optimization included an energy change of $<5 \times 10^{-6}$ eV atom^{-1} between steps, a maximal force of <0.008 eV Å^{-1} and a maximal component of the stress tensor <0.02 GPa. Phonon frequencies were obtained from density functional perturbation theory (DFPT) calculations.[24,25] Raman intensities were computed using DFPT with the "$2n+1$" theorem approach.[26]

2. Results

2.1. *Single-crystal synchrotron X-ray diffraction at 40(2) GPa*

In order to determine the crystal structure of the unknown phase observed by Raman spectroscopy after laser-heating the TeO_2 + CO_2 mixture at 40(2) GPa, we employed synchrotron X-ray diffraction at beam line P02.2. First, we collected X-ray diffraction data on a 2D grid across the sample chamber using a spot size of $\approx 2 \times 2$ μm^2 in order to locate promising positions for the collection of single-crystal diffraction data. Afterwards, we employed synchrotron single-crystal X-ray diffraction on selected locations of the grid where unidentified reflections were present. Fig. S 1 a shows a part of the reciprocal space reconstruction for the $(h\bar{1}l)$ plane of the single-crystal on which the crystal structure at 40(2) GPa was determined.

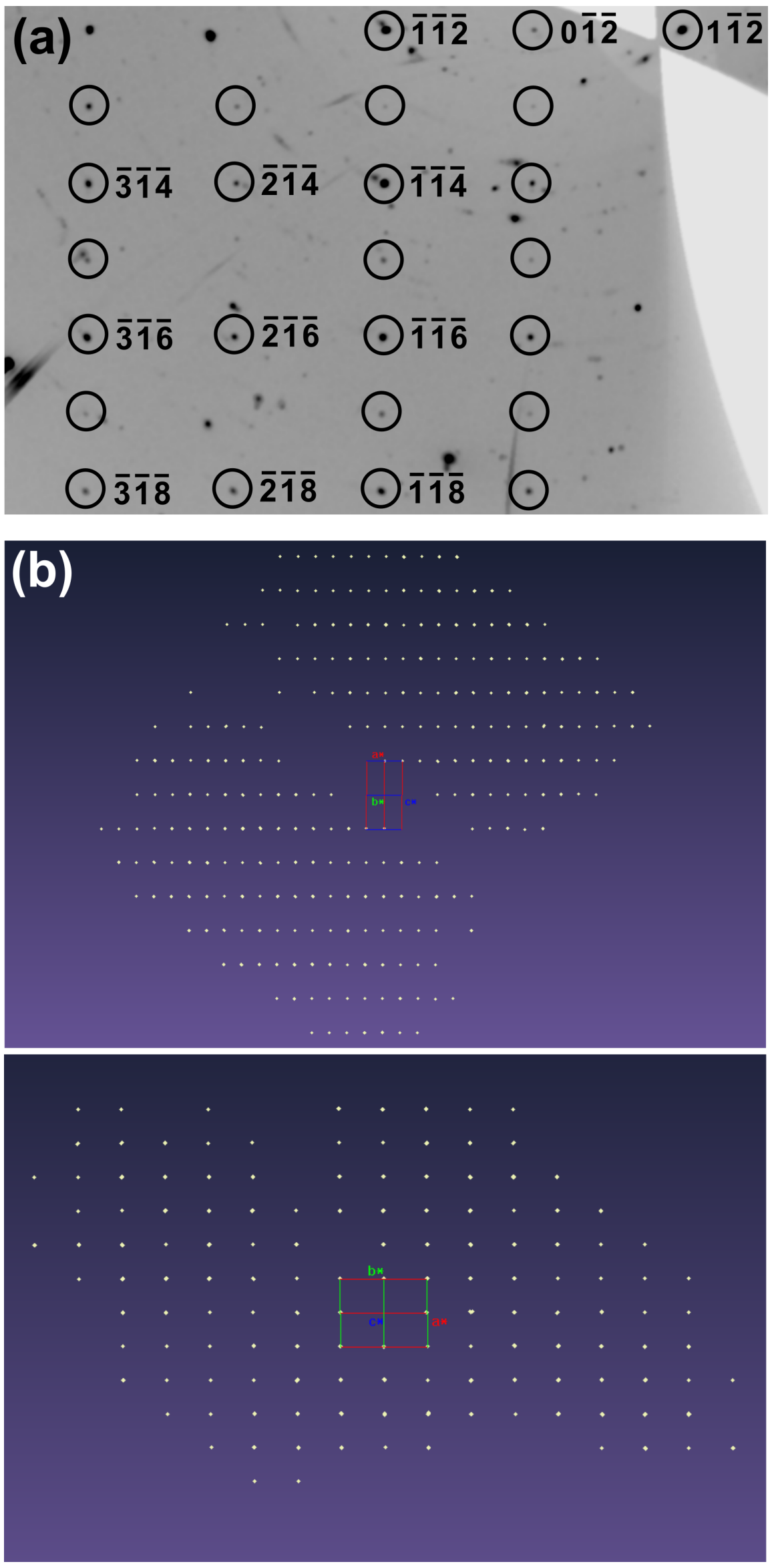

Figure S 1: (a) Reciprocal space reconstruction for the $(h\bar{1}l)$ plane of Te[CO_3]O at 40(2) GPa. (b) Schematic depiction of the reflections in reciprocal space using the Ewald-Explorer in CrysAlis after data reduction which were later used for the refinement. Projections of the reciprocal space are shown along b^* (top) and c^* (bottom).

In addition to the reflections of the unknown phase, we observed reflections and powder rings of CO_2-V, of TeO_2 and of diamond in the collected diffraction data (Fig. S 1 a). Fig. S 1 b shows the projection of the reciprocal space along b^* (top) and c^* (bottom). The effect of the shading of diffracted beams due to the metallic body of the DAC can be observed.

We solved the crystal structure of the unknown phase from the single crystal diffraction data collected at 40(2) GPa in the monoclinic space group $P2_1/c$ (No. 14) with $Z = 4$ and Te[CO_3]O composition (Table S 1). The low R_1-value of (3.0%) in combination with a high reflection to parameter ratio (10.5:1) for a DAC experiment reveal a convincing structure refinement. Moreover, the atomic displacement parameters of the light carbon and oxygen atoms could be refined anisotropically next to the heavy tellurium atoms, confirming the very high quality of the collected diffraction data. In addition, no constraints or restraints had to be introduced for the structure refinement and the experimental crystallographic parameters at 40(2) GPa are in agreement with the data derived from our DFT calculations (Table S 1).

Table S 1: Structural parameters of Te[CO_3]O-$P2_1/c$ at 40(2) GPa from single-crystal structure solution (ambient temperature) in comparison to data from DFT calculations at the same pressure (athermal limit).

	**Single Crystal**	**DFT**
**Crystal data**		
Crystal system	monoclinic	monoclinic
Space group	$P2_1/c$	$P2_1/c$
Chemical formula	Te[CO_3]O	Te[CO_3]O
M_r	203.61	203.61
a (Å)	4.3690(3)	4.3507
b (Å)	5.606(2)	5.7637
c (Å)	8.4270(8)	8.3648
α (°)	90.0	90.0
β (°)	90.32(1)	90.0003
γ (°)	90.0	90.0
V (Å^3)	206.41(7)	208.74
Z	4	4
**Data collection**		-
F_{000}	360	-
θ range (°)	1.91–18.11	-
measured reflections	1000	-
independent reflections	577	-
reflections $I > 2\sigma(I)$	494	-
R_{int}	0.013	-
		-
**Refinement**		-
$R_1[I > 2\sigma(I)]$, $wR_2(I)$	0.030, 0.080	-
No. of reflections	577	-
No. of parameters	55	-
No. of restraints	0	-
No. of constraints	0	-
$\Delta\rho_{max}$, $\Delta\rho_{min}$ (e Å^{-3})	2.31, −1.90	-

2.2. *Single-crystal synchrotron X-ray diffraction at 20(2) GPa*

The crystal structure of the unknown phase observed by Raman spectroscopy after laser-heating the TeO_2 + CO_2 mixture at lower pressures (20(2) GPa) was determined using the same approach than for Te[CO_3]O at higher pressures. Synchrotron X-ray diffraction was employed at beam line ID11 using a spot size of $\approx 0.6 \times 0.6$ µm^2. Fig. S 2 a shows a part of the reciprocal space reconstruction for the $(h\bar{1}l)$ plane of the single-crystal on which the crystal structure of the unknown phase at 20(2) GPa was determined.

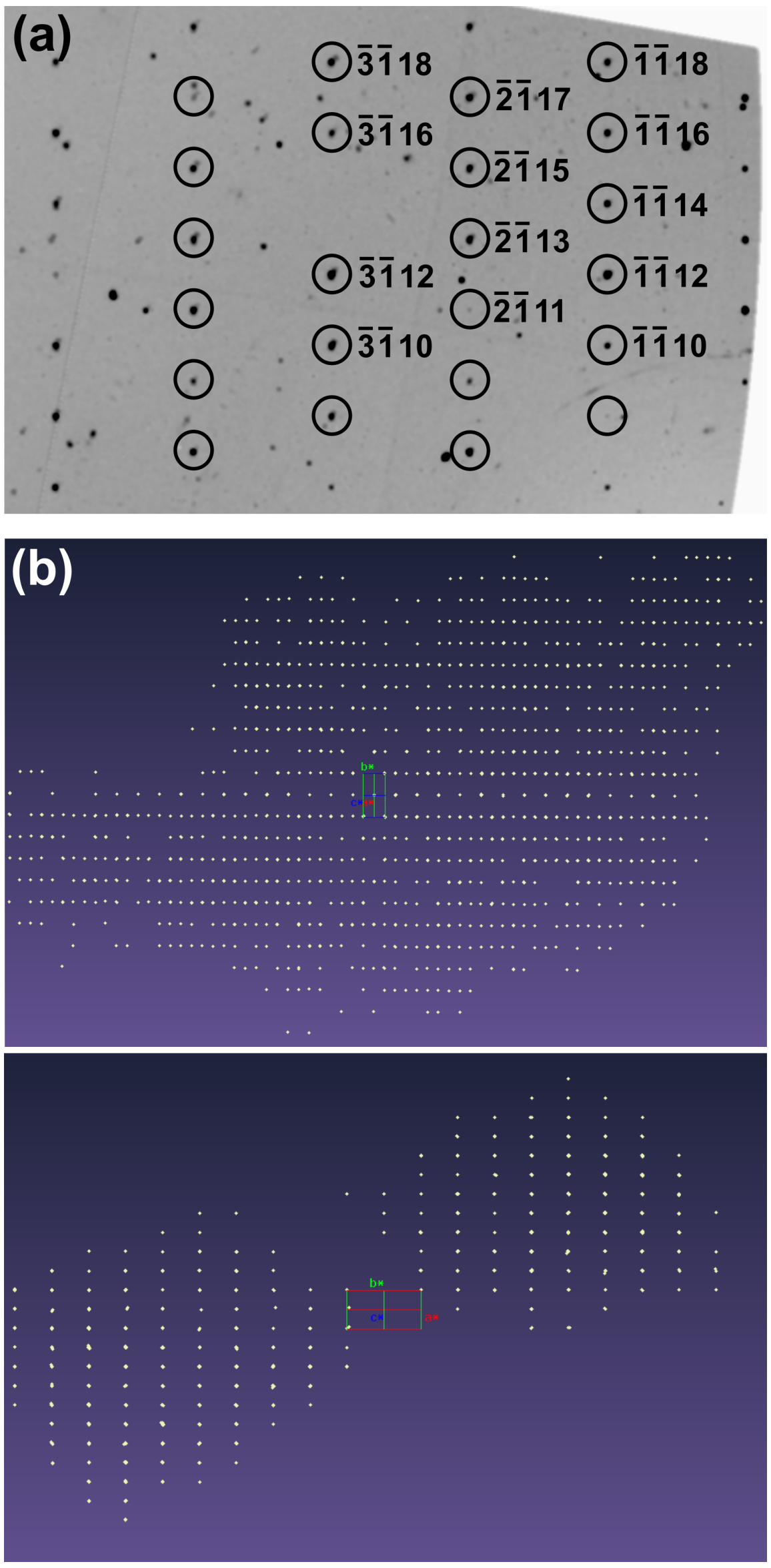

Figure S 2: (a) Reciprocal space reconstruction for the $(h\bar{1}l)$ plane of $Te_4O_7[CO_3]$ at 20(2) GPa. (b) Schematic depiction of the reflections in reciprocal space using the Ewald-Explorer in CrysAlis after data reduction which were later used for the refinement. Projections of the reciprocal space are shown along a^* (top) and c^* (bottom).

Besides the reflection of the unknown phase, reflections and powder rings of different CO_2 polymorphs (III, II, IV), of TeO_2 and of diamond could be observed in the collected diffraction data (Fig. S 2 a). The effect of the shading of diffracted beams due to the metallic body of the DAC and the effect of the inactive detector-areas due to the segments of the Eiger2 X detector can be observed in the projection of the reciprocal space (Fig. S 1 b).

The crystal structure of the unknown phase was solved from the single crystal diffraction data collected at 20(2) GPa in the orthorhombic space group *Iba*2 (No. 45) with $Z = 8$ and $Te_4O_7[CO_3]$ composition (Table S 2). The low R_1-value of (3.3%) in combination with a very high reflection to parameter ratio (15.8:1) for a DAC experiment reveal a very convincing structure refinement. Similar to the structure refinement of Te[CO_3]O the atomic displacement parameters of the light carbon and oxygen atoms could be refined anisotropically next to the heavy tellurium atoms. No constraints or restraints had to be introduced for the structure refinement. The experimental crystallographic parameters of $Te_4O_7[CO_3]$ at 20(2) GPa are in agreement with the data derived from our DFT calculations (Table S 2). We tested the experimental structural model of $Te_4O_7[CO_3]$ using the *PLATON*/checkCIF program, which does not suggests a higher space group symmetry or a centrosymmetric crystal structure.[27] This is consistent with our DFT-based geometry optimization which retain the acentric space group symmetry.

Table S 2: Structural parameters of $Te_4O_7[CO_3]$-*Iba*2 at 20(2) GPa from single-crystal structure solution (ambient temperature) in comparison to data from DFT calculations at the same pressure (athermal limit).

	**Single Crystal**	**DFT**
**Crystal data**		
Crystal system	orthorhombic	orthorhombic
Space group	*Iba*2	*Iba*2
Chemical formula	$Te_4O_7[CO_3]$	$Te_4O_7[CO_3]$
M_r	682.41	682.41
a (Å)	21.576(1)	22.1455
b (Å)	10.671(2)	10.7026
c (Å)	5.5405(2)	5.5299
α (°)	90.0	90.0
β (°)	90.0	90.0
γ (°)	90.0	90.0
V (Å^3)	1275.6(2)	1310.67
Z	8	8
**Data collection**		-
F_{000}	2352	-
θ range (°)	1.82–18.70	-
measured reflections	2910	-
independent reflections	2167	-
reflections $I > 2\sigma(I)$	2024	-
R_{int}	0.022	-
		-
**Refinement**		-
$R_1[I > 2\sigma(I)]$, $wR_2(I)$	0.033, 0.087	-
No. of reflections	2167	-
No. of parameters	137	-
No. of restraints	0	-
No. of constraints	0	-
$\Delta\rho_{max}$, $\Delta\rho_{min}$ (e Å^{-3})	2.56, −1.54	-

2.3. Compressibility of Te[CO$_3$]O and Te$_4$O$_7$[CO$_3$]

We used our DFT-based calculations to derive the p, V-relation for Te[CO$_3$]O between 50 GPa and ambient pressure. Our DFT-calculations predict that Te[CO$_3$]O is undergoing an isostructural phase transition during decompression. In order to obtain the bulk modulus (K_0) and its pressure derivative (K_p) for the high- and low-pressure phase of Te[CO$_3$]O we fitted separate 3rd-order Birch-Murnaghan equation of states (EoS) to unit cell volumes obtained for the different polymorphs (Fig. S 3).[28,29] We employed the software package EOSFit7-GUI for the fit.[30] The theoretical bulk modulus of the high-pressure phase is $K_0 = 80.0(6)$ GPa with $K_\mathrm{p} = 5.76(4)$, while it is $K_0 = 32.4(6)$ GPa with $K_\mathrm{p} = 6.2(1)$ for the low-pressure phase of Te[CO$_3$]O.

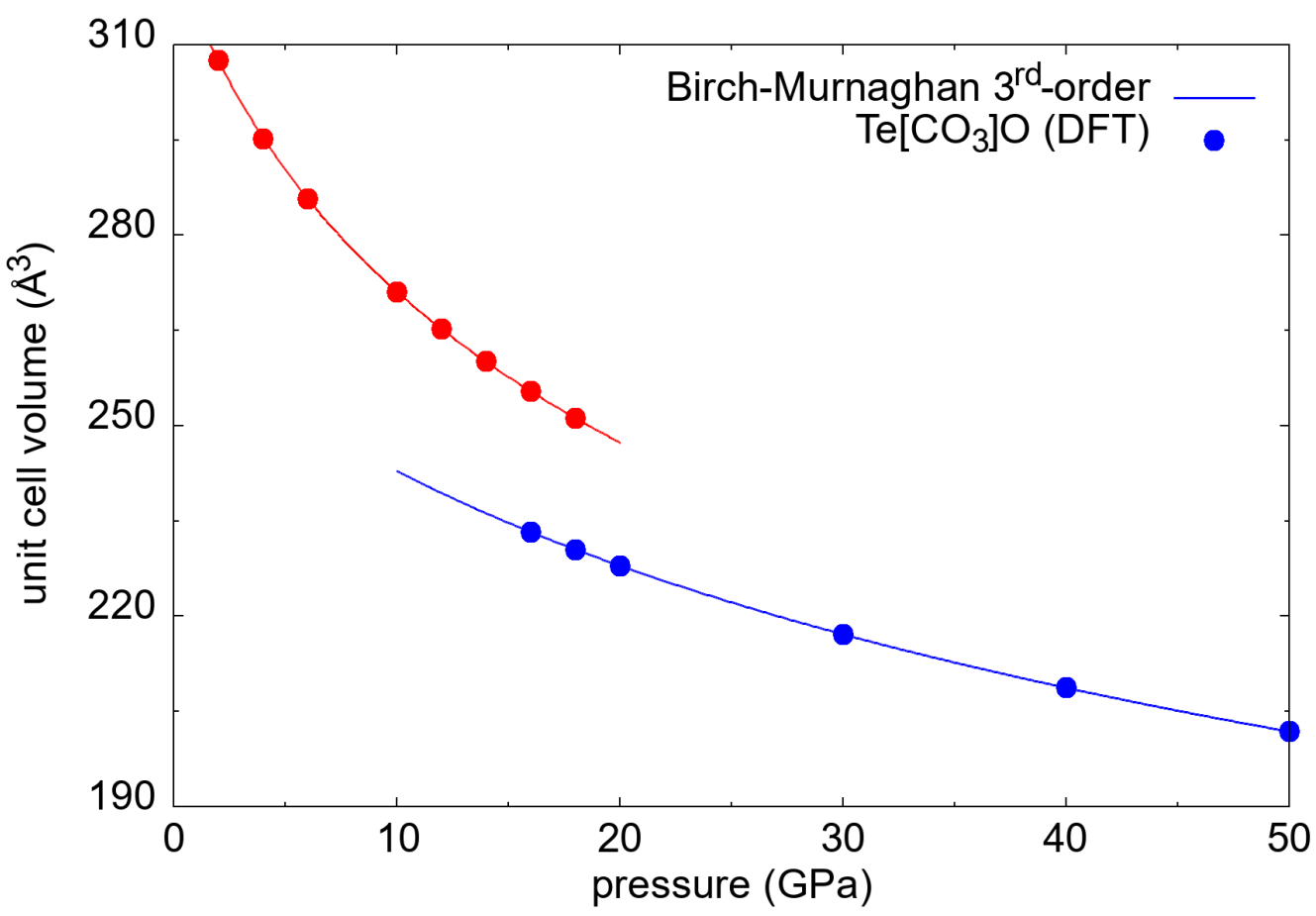

Figure S 3: Separate 3rd-order Birch-Murnaghan EoS are fitted to the p, V-data of the high- (16–50 GPa) and low-pressure phase (2–18 GPa) of Te[CO$_3$]O obtained by the DFT-based calculations.

In addition, we used our DFT-based calculations to derive the p, V-relation for Te$_4$O$_7$[CO$_3$] (Fig. S 4). The bulk modulus (K_0) and its pressure derivative (K_p) were derived in a similar manner than for Te[CO$_3$]O. The theoretical bulk modulus of Te$_4$O$_7$[CO$_3$] is $K_0 = 51(4)$ GPa with $K_\mathrm{p} = 6.7(4)$.

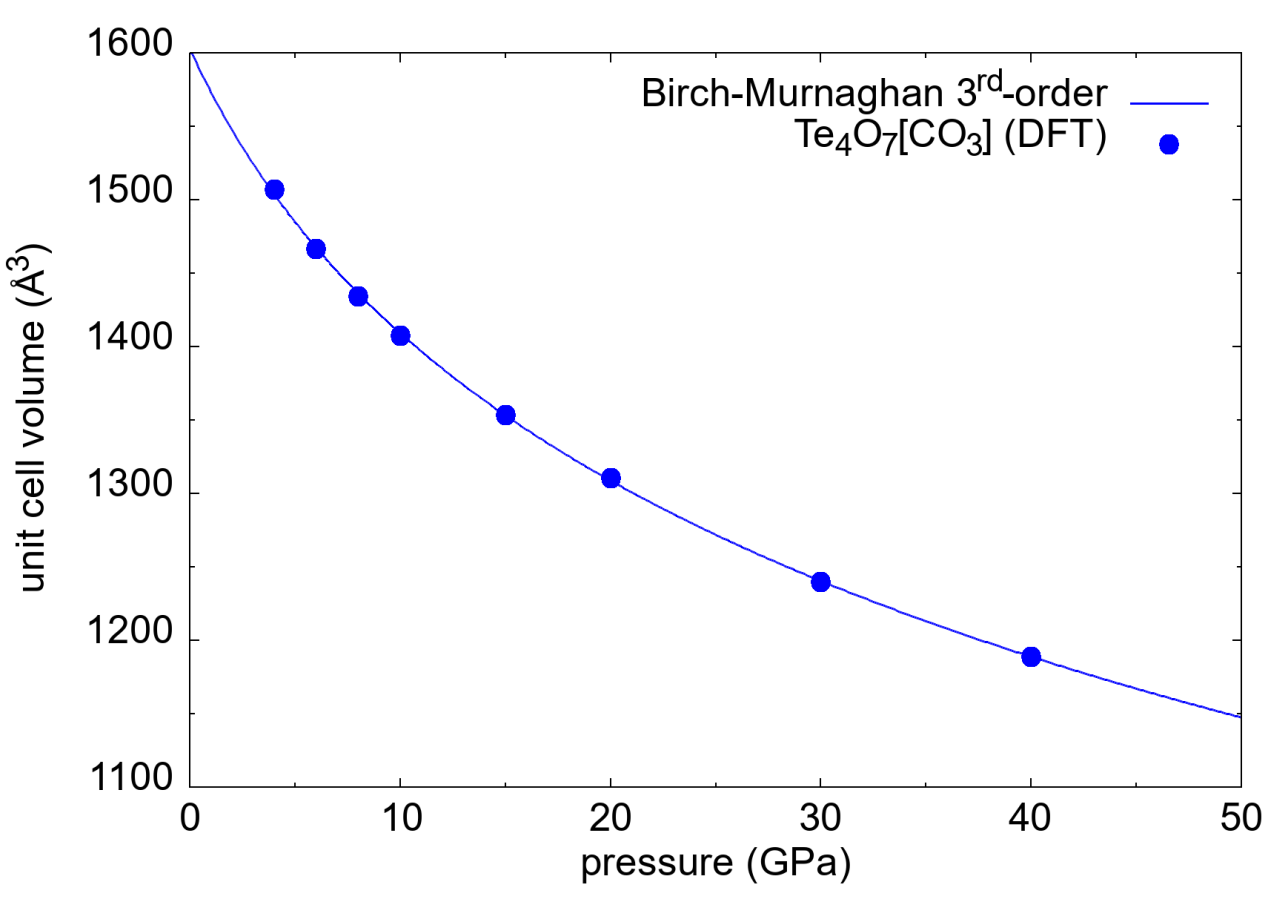

Figure S 4: A 3rd-order Birch-Murnaghan EoS is fitted to the p, V-data of Te$_4$O$_7$[CO$_3$] (4–40 GPa) obtained by the DFT-based calculations.

References

(1) Boehler, R. New diamond cell for single–crystal X-ray diffraction. *Rev. Sci. Instrum.* **2006**, *77*, 115103, DOI: 10.1063/1.2372734

(2) Akahama, Y.; Kawamura, H.; Pressure calibration of diamond anvil Raman gauge to 310 GPa. *J. Appl. Phys.* **2006**, *100*, 043516, DOI: 10.1063/1.2335683

(3) Yoo, C. S.; Cynn, H.; Gygi, F.; Galli, G.; Iota, V.; Nicol, M.; Carlson, S.; Häusermann, D.; Mailhiot, C. Crystal Structure of Carbon Dioxide at High Pressure: "Superhard" Polymeric Carbon Dioxide. *Phys. Rev. Lett.* **1999**, *83*, 5527–5530, DOI: 10.1103/PhysRevLett.83.5527

(4) Spahr, D.; Bayarjargal, L.; Brüning, L.; Kovalev, V.; Bykova, E.; Bykov, M.; Milman, V.; Mezouar, M.; Winkler, B. Synthesis and Crystal Structure of Anhydrous Di-iodyl Carbonate $(IO_2)_2[CO_3]$, Hosting I^{5+}-Cations. *JACS Au* **2025**, *5*, 4675–4680, DOI: 10.1021/jacsau.5c00829

(5) Bayarjargal, L.; Fruhner, C.-J.; Schrodt, N.; Winkler, B. $CaCO_3$ phase diagram studied with Raman spectroscopy at pressures up to 50 GPa and high temperatures and DFT modeling. *Phys. Earth Planet. Inter.* **2018**, *281*, 31–45, DOI: 10.1016/j.pepi.2018.05.002

(6) Benedetti, L. R.; Loubeyre, P. Temperature gradients, wavelength-dependent emissivity, and accuracy of high and very-high temperatures measured in the laser-heated diamond cell. *High Press. Res.* **2004**, *24*, 423–455, DOI: 10.1080/08957950412331331718

(7) Du, Z.; Amulele, G.; Benedetti, L. R.; Lee, K. K. M. Mapping temperatures and temperature gradients during flash heating in a diamond-anvil cell. *Rev. Sci. Instrum.* **2013**, *84*, 075111, DOI: 10.1063/1.4813704

(8) Wojdyr, M. *Fityk*: a general-purpose peak fitting program. *J. Appl. Cryst.* **2010**, *43*, 1126–1128, DOI: 10.1107/S0021889810030499

(9) Liermann, H.-P.; Konôpková, Z.; Morgenroth, W.; Glazyrin, K.; Bednarčik, J.; McBride, E. E.; Petitgirard, S.; Delitz, J. T.; Wendt, M.; Bican, Y.; Ehnes, A.; Schwark, I.; Rothkirch, A.; Tischer, M.; Heuer, J.; Schulte-Schrepping, H.; Kracht, T.; Franz, H. The Extreme Conditions Beamline P02.2 and the Extreme Conditions Science Infrastructure at PETRAIII. *J. Synchrotron Radiat.* **2014**, *22*, 908–924, DOI: 10.1107/S1600577515005937

(10) Wright, J.; and Giacobbe, C.; and Majku, M. New opportunities at the Materials Science Beamline at ESRF to exploit high energy nano-focus X-ray beams. *Curr. Opin. Solid. St. M.* **2020**, *24*, 100818, DOI: 10.1016/j.cossms.2020.100818

(11) Prescher, C.; Prakapenka, V. B. *DIOPTAS*: a program for reduction of two-dimensional X-ray diffraction data and data exploration. *High. Press. Res.* **2015**, *35*, 223–230, DOI: 10.1080/08957959.2015.1059835

(12) Agilent, CrysAlis PRO, Yarnton, England, **2014**

(13) Aslandukov, A.; Aslandukov, M.; Dubrovinskaia, N.; Dubrovinsky, L. *Domain Auto Finder* (*DAFi*) program: the analysis of single-crystal X-ray diffraction data from polycrystalline sample. *J. Appl. Cryst.* **2022**, *55*, 1383–1391, DOI: 10.1107/S1600576722008081

(14) Dolomanov, O. V.; Bourhis, L. J.; Gildea, R. J.; Howard, J. A. K.; Puschmann, H. *OLEX2*: a complete structure solution, refinement and analysis program. *J. Appl. Cryst.* **2009**, *42*, 339–341, DOI: 10.1107/S0021889808042726

(15) Sheldrick, G. M. *SHELXT* — Integrated space-group and crystal-structure determination. *Acta. Cryst.* **2015**, *A71*, 3–8, DOI: 10.1107/S2053273314026370

(16) Sheldrick, G. M. Crystal structure refinement with *SHELXL*. *Acta. Cryst.* **2015**, *C71*, 3–8, DOI: 10.1107/S2053229614024218

(17) Hohenberg, P.; Kohn, W. Inhomogeneous Electron Gas. *Phys. Rev.* **1967**, *136*, B864–B871, DOI: 10.1103/PhysRev.136.B864

(18) Perdew, J. P.; Burke, K.; Ernzerhof, M. Generalized Gradient Approximation Made Simple. *Phys. Rev. Lett.* **1996**, *77*, 3865–3868, DOI: 10.1103/PhysRevLett.77.3865

(19) Clark, S. J.; Segall, M. D.; Pickard, C. J.; Hasnip, P. J.; Probert, M. I. J.; Refson, K.; Payne, M. C. First principles methods using CASTEP. *Z. Kristallogr.* **2005**, *220*, 567–570, DOI: 10.1524/zkri.220.5.567.65075

(20) BIOVIA, Materials Studio, San Diego, USA, **2025**

(21) Lejaeghere, K.; Bihlmayer, G.; Björkman, T.; Blaha, P.; Blügel, S.; Blum, V.; Caliste, D.; Castelli, I. E.; Clark, S. J.; Dal Corso, A. et al. Reproducibility in density functional theory calculations of solids. *Science* **2016**, *351*, aad3000, DOI: 10.1126/science.aad3000

(22) Tkatchenko, A.; Scheffler, M. Accurate Molecular Van Der Waals Interactions from Ground-State Electron Density and Free-Atom Reference Data. *Phys. Rev. Lett.* **2009**, *102*, 073005, DOI: 10.1103/PhysRevLett.102.073005

(23) Monkhorst, H. J.; Pack, J. D. Special points for Brillouin-zone integrations. *Phys. Rev. B* **1976**, *13*, 5188–5192, DOI: 10.1103/PhysRevB.13.5188

(24) Baroni, S.; de Gironcoli, S.; Dal Corso, A.; Giannozzi, P. Phonons and related crystal properties from density-functional perturbation theory. *Rev. Mod. Phys.* **2001**, *73*, 515–562, DOI: 10.1103/RevModPhys.73.515

(25) Refson, K.; Tulip, P. R.; Clark, S. J. Variational density-functional perturbation theory for dielectrics and lattice dynamics. *Phys. Rev. B* **2006**, *73*, 155114, DOI: 10.1103/PhysRevB.73.155114

(26) Miwa, K. Prediction of Raman spectra with ultrasoft pseudopotentials. *Phys. Rev. B* **2011**, *84*, 094304, DOI: 10.1103/PhysRevB.84.094304

(27) Spek, A. L. Single-crystal structure validation with the program *PLATON*. *J. Appl. Cryst.* **2003**, *36*, 7–13, DOI: 10.1107/S0021889802022112

(28) Murnaghan, F. The Compressibility of Media under Extreme Pressures. *Proc. Natl. Acad. Sci.* **1944**, *30*, 244–247, DOI: 10.1073/pnas.30.9.244

(29) Birch, F. Finite Elastic Strain of Cubic Crystals. *Phys. Rev.* **1947**, *71*, 809–824, DOI: 10.1103/PhysRev.71.809

(30) Gonzalez-Platas, J.; Alvaro, M.; Nestola, F.; Angel, R. *EosFit7-GUI*: a new graphical user interface for equation of state calculations, analyses and teaching. *J. Appl. Cryst.* **2016**, *49*, 1377–1382, DOI: 10.1107/S1600576716008050
